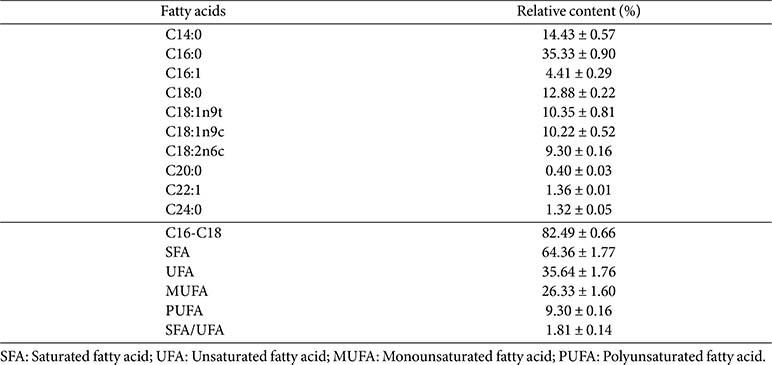# Sustainable PUFA-Rich Lipid-Accumulating Biomass Production via Dual Waste Valorization Using Heterotrophic Microalgae Cultivated on Anaerobic Effluent and Molasses

**DOI:** 10.4014/jmb.2506.06037

**Published:** 2025-10-28

**Authors:** Wageeporn Maneechote, Wasu Pathom-aree, Apiwit Kamngoen, Antira Wichaphian, Benjamas Cheirsilp, Kuan Shiong Khoo, Shuhao Huo, Piroonporn Srimongkol, Sirasit Srinuanpan

**Affiliations:** 1 Microbial Biorefinery and Biochemical Process Engineering Research Group, Chiang Mai University, Chiang Mai 50200, Thailand; 2 Department of Biology, Faculty of Science, Chiang Mai University, Chiang Mai 50200, Thailand; 3 Office of Research Administration, Office of the University, Chiang Mai University, Chiang Mai 50200, Thailand; 4 Center of Excellence in Microbial Diversity and Sustainable Utilization, Faculty of Science, Chiang Mai University, Chiang Mai 50200, Thailand; 5 Center of Excellence in Innovative Biotechnology for Sustainable Utilization of Bioresources, Faculty of Agro- Industry, Prince of Songkla University, Hat Yai, Songkhla, 90110, Thailand; 6 Department of Chemical Engineering and Materials Science, Yuan Ze University, Taoyuan, Taiwan; 7 School of Food and Biological Engineering, Jiangsu University, Zhenjiang 212013, China; 8 High-Value Food from Mushrooms and Bioactive Plants in the Green Economy Value Chain Research Group, Institute of Biotechnology and Genetic Engineering, Chulalongkorn University, 254 Phayathai Road, Pathumwan, Bangkok 10330, Thailand

**Keywords:** Anaerobic effluent, molasse, heterotrophic, microalgae, lipid, PUFA

## Abstract

The growing global demand for sustainable solutions necessitates innovative strategies for biomass and high-value compound production from waste. This study explores the heterotrophic cultivation of microalga *Chlorella* sp. G049 using anaerobic effluent (AE) derived from food waste and molasses as cost-effective substrates to produce polyunsaturated fatty acid (PUFA)-rich lipids while concurrently treating wastewater. Initially, one-factor-at-a-time (OFAT) experiments identified 12.5% AE and 20 g/l molasses as effective concentrations for promoting growth. Subsequently, Response Surface Methodology (RSM) was employed to optimize co-utilization of AE and molasses, maximizing biomass concentration, lipid yield, and PUFA content. The optimal conditions—18.5% AE and 18.18 g/l molasses—resulted in significantly enhanced biomass concentration (4.09 g/l), lipid yield (87.00 mg/l), and PUFA content (9.30%). The extracted lipids exhibited nutritional potential and met international biodiesel standards, with a notably high cetane number (62.95) and oxidative stability (15.27 h). Additionally, the microalgal system effectively removed chemical oxygen demand (91.79%), ammonium (63.19%), nitrate (73.21%), and phosphate (96.76%), confirming its robust wastewater remediation capacity. This dual-purpose approach points out the advantages of valorizing waste streams into value-added bioproducts and supports the advancement of circular bioeconomy principles.

## Introduction

The escalating global demand for sustainable energy systems and the imperative to transition towards circular economy principles have significantly intensified research interest in microalgae. This urgency is driven by pressing environmental concerns such as climate change, the depletion of fossil fuel reserves, and the escalating generation of various waste streams [1]. These photosynthetic microorganisms are increasingly recognized as versatile biological platforms for producing a wide range of products, including bulk biofuels (*e.g.*, biodiesel, bioethanol, and biohydrogen) and high-value compounds (*e.g.*, omega-3 fatty acids, carotenoids, pigments, antioxidants, and protein-rich animal feed) [2]. Microalgae offer distinct advantages over traditional agricultural feedstocks, such as terrestrial crops, due to their remarkably rapid growth rates, minimal land footprint, and impressive efficiency in accumulating lipids [3]. Their unique metabolic pathways also enable them to synthesize essential polyunsaturated fatty acids (PUFAs), which are crucial for human nutrition and animal health, offering a sustainable alternative to traditional marine sources [4]. However, despite their considerable promise, the widespread economic viability of microalgal biotechnology currently faces substantial challenges, primarily stemming from the high operational costs associated with conventional synthetic growth media and the inherently energy-intensive nature of traditional cultivation and downstream processing systems (*e.g.*, mixing, harvesting, and dewatering) [1].

Traditional photoautotrophic cultivation, which relies on light as the primary energy source, incurs significant energy expenditures, particularly for artificial illumination in controlled photobioreactors, contributing substantially to overall production costs [4]. Moreover, this method faces inherent limitations, including restricted light penetration in dense cultures, which often leads to photooxidative stress in surface cells and light starvation for cells deeper within the culture, thereby limiting achievable cell densities [5]. Furthermore, their dependency on atmospheric CO_2_ for carbon fixation can be a bottleneck for rapid growth unless external CO_2_ supplementation is provided [1]. These constraints have spurred considerable research into heterotrophic cultivation, an alternative and highly promising approach where microalgae utilize various organic carbon sources in the complete absence of light [4]. This method holds the profound potential to achieve significantly higher cell densities and biomass productivities—often an order of magnitude greater than photoautotrophic systems—while simultaneously reducing energy costs associated with lighting and minimizing the risks of photoinhibition [6]. Nevertheless, the successful and economically competitive implementation of heterotrophic cultivation is critically dependent on the identification and consistent availability of abundant, inexpensive, and readily assimilable carbon and nutrient sources [2].

A particularly appealing and ecologically sound strategy to address both cultivation costs and the pressing need for sustainable waste management is the valorization of diverse industrial and agricultural waste streams as growth substrates [1]. Anaerobic effluent (AE), derived from the anaerobic digestion of food waste or other organic matter, for instance, is a rich source of essential macronutrients such as nitrogen (in the form of ammonia and nitrates), phosphorus (as phosphates), and various vital trace elements [7]. Although AE is classified as a waste stream, its utilization as a cultivation substrate can serve a dual purpose: reducing environmental burden through partial nutrient removal and enabling the production of biomass for downstream valorization. This approach does not aim to achieve complete wastewater treatment but rather to integrate waste management with resource recovery in a cost-effective manner. Concurrently, industrial by-products like molasses, a concentrated syrup left over from sugar refining, offer readily available, inexpensive, and abundant sources of organic carbon, primarily in the form of sucrose, glucose, and fructose [8]. While numerous recent studies have compellingly demonstrated the potential of diverse waste streams for supporting microalgal cultivation [7], the intricate nuances of optimizing multi-substrate systems—especially understanding the complex interactive and often synergistic or antagonistic effects of different waste components on critical parameters like biomass accumulation, total lipid biosynthesis, and the precise fatty acid composition—remain largely unexplored and poorly understood. Such interactions can significantly influence metabolic pathways and overall productivity.

The fatty acid profile of microalgal lipids is a critical determinant for both their potential nutritional value and their suitability for biofuel applications. From a nutritional perspective, specific PUFAs (*e.g.*, omega-3 fatty acids like eicosapentaenoic acid (EPA) and docosahexaenoic acid (DHA), and omega-6 fatty acids like linoleic acid) are recognized for their profound health benefits, including reducing the risk of cardiovascular disease, exerting potent anti-inflammatory properties, and supporting cognitive and neurological development [4, 9, 10]. These benefits stem from their role in cell membrane structure and as precursors for signaling molecules. For biodiesel production, an optimal fatty acid composition is crucial for balancing key fuel properties such as a high cetane number (indicating good ignition quality), excellent oxidative stability (preventing degradation during storage), and favorable cold flow characteristics (ensuring operability at low temperatures) [11]. The existing scientific literature presents somewhat contradictory findings regarding the precise impact of heterotrophic conditions on microalgal fatty acid profiles. Some studies report an increase in saturated fatty acid (SFA) content under heterotrophic growth, which is generally desirable for enhancing biodiesel properties like cetane number and oxidative stability [12]. Conversely, other investigations indicate the maintenance or even enhancement of high PUFA levels, making them highly suitable for nutritional products [4, 13]. This divergence underscores the need for a more comprehensive and nuanced understanding of how specific cultivation parameters, including nutrient ratios and organic carbon type, influence lipid quality and fatty acid desaturation pathways.

Furthermore, the optimization of complex multi-substrate cultivation systems has historically relied on traditional one-factor-at-a-time (OFAT) experimental designs. This conventional approach, however, suffers from significant limitations: it assesses the effect of only one variable at a time while keeping others constant, inherently failing to capture the intricate synergistic and antagonistic interactions that can occur between multiple factors. This often leads to suboptimal conditions and an incomplete understanding of the system's true potential [14]. Response Surface Methodology (RSM), a more sophisticated and statistically rigorous technique, offers a robust framework for the simultaneous optimization of multiple responses [4, 9, 12]. By building empirical models that describe the relationship between inputs and outputs, RSM enables a more comprehensive understanding of the system, including identifying optimal factor combinations and visualizing interaction effects [15]. Despite its clear advantages, its application in waste-based microalgal cultivation systems, especially for achieving multi-objective optimization (*e.g.*, simultaneously maximizing biomass, lipid yield, and specific fatty acid compositions), is still remarkably limited. The overarching challenge lies in developing precise cultivation strategies that can concurrently maximize volumetric biomass production, enhance total lipid accumulation, and maintain desirable fatty acid compositions tailored for specific end-use applications, often requiring a delicate balance of metabolic trade-offs within the microalgal cell.

This study focuses on developing a sustainable waste-based heterotrophic cultivation system using AE and molasses to recover nutrients and enhance *Chlorella* sp. G049 biomass and lipid production. As the process was conducted in dark conditions, CO_2_ fixation was not the primary goal; instead, the work aims to valorize waste streams while enabling potential downstream biofuel applications, aligning with the principles of a circular bioeconomy. To achieve this, our specific objectives include: (1) systematically evaluating the individual effects of varying anaerobic effluent and molasses concentrations on the heterotrophic growth performance of *Chlorella* sp. G049; (2) applying RSM to meticulously optimize multi-substrate cultivation parameters for the simultaneous maximization of biomass concentration, total lipid yield, and desired PUFA content; (3) comprehensively characterizing the nutritional quality and assessing the precise biodiesel potential of the lipids produced under optimized conditions; and (4) critically assessing the wastewater treatment capacity of the optimized microalgal cultivation system, quantifying its efficiency in nutrient removal.

## Materials and Methods

### Microalgae Strain and Pre-Cultivation

The microalga *Chlorella* sp. G049, which was used in this study, was obtained from the Algal and Cyanobacterial Research Laboratory, Department of Biology, Faculty of Science, Chiang Mai University, Thailand. It was selected for this study due to its robust growth, ease of cultivation, adaptability to nutrient-rich and wastewater-derived media, and demonstrated suitability for biomass and metabolite production in our previous study [4, 9, 10, 12]. Cultivation commenced using Jaworski’s Medium (JM) with the following composition: Ca(NO_3_)2·4H_2_O 0.02 g/l, KH_2_PO_4_ 0.0124 g/l, MgSO_4_·7H_2_O 0.05 g/l, NaHCO_3_ 0.0159 g/l, EDTA-FeNa 0.00225 g/l, EDTANa_2_ 0.00225 g/l, H_3_BO_3_ 0.00248 g/l, MnCl_2_·4 H_2_O 0.00139 g/l, (NH_4_)_6_Mo_7_O_24_·4H_2_O 0.001 g/l, cyanocobalamin 0.004 g/l, thiamine HCl 0.04 mg/l, biotin 0.04 mg/l, NaNO_3_ 0.08 g/l, and Na_2_HPO_4_·12H_2_O 0.036 g/l [12]. The initial pH of the medium was adjusted to 7.0. Seed cultures were cultivated in 400 ml of JM medium contained within 500 ml flasks, with continuous aeration at a flow rate of 6.5 L/min. The cultures were maintained at 25 ± 2°C under continuous illumination with cool-white, fluorescent light at an intensity of 70 μmol/m^2^/s. Cultivation was continued until the stationary growth phase was reached, typically within 7 to 10 days.

### Anaerobic Effluent Source and Preparation

Anaerobic effluent (AE) derived from anaerobic digestion of food waste was obtained from the Energy Research and Development Institute of Nakornping, Chiang Mai University, Thailand. The raw effluent had a pH of 7.8 and contained chemical oxygen demand (COD) of 2,144 mg/l, ammonium-nitrogen (NH_4_^+^-N) at 352 mg/l, nitrate-nitrogen (NO_3_^-^-N) at 51 mg/l, phosphate-phosphorus (PO_4_^3-^-P) at 84 mg/l, sodium (Na) at 101.38 mg/l, calcium (Ca) at 12.43 mg/l, magnesium (Mg) at 5.90 mg/l, sulfur (S) at 0.084 mg/l, iron (Fe) at 0.41 mg/l, copper (Cu) at 0.008 mg/l, and boron (B) at 0.078 mg/l. Prior to use, the effluent was filtered through Whatman No. 1 filter paper to remove solid particles and was then stored at 4°C for future use.

### Heterotrophic Cultivation of *Chlorella* sp. G049 Using Anaerobic Effluent (AE)

*Chlorella* sp. G049 was cultivated in 250 ml flasks containing 100 ml of AE, supplemented with varying concentrations (6.25%, 12.5%, 25%, 50%, and 100%). For comparison, cultivation in Jaworski’s Medium (JM) was also conducted under identical conditions. The microalgal preculture was inoculated into each flask to achieve an initial cell density of 0.15 × 10^8^ cells/ml. To ensure heterotrophic conditions, the culture flasks were wrapped in aluminum foil to prevent light exposure. Cultivation was performed on an orbital shaker at 120 rpm and 25°C for 6 days. Cell dry weight and pH were measured, and the AE concentration that yielded the highest biomass was selected for subsequent experiments.

### Heterotrophic Cultivation of *Chlorella* sp. G049 Using Molasses

*Chlorella* sp. G049 was cultivated in 250 ml flasks containing 100 ml of optimized AE medium (as determined in Section 2.3), supplemented with varying concentrations of molasses (from the Aung Peang Heang Agriculture Store in Thailand): 1, 5, 10, 15, 20, 25, and 30 g/l. At the high concentration of raw molasses (30 g/l), the COD was 5,775 mg/l, NH_4_^+^-N was 205 mg/l, NO_3_^-^-N was 21 mg/l, PO_4_^3-^-P was 19.2 mg/l, and total sugar was 3,550 mg/l. The mineral contents were as follows: Na, 14.79 mg/l; Ca, 48.86 mg/l; Mg, 70.52 mg/l; S, 0.84 mg/l; Fe, 1.82 mg/l; Mn, 0.86 mg/l; Cu, 0.027 mg/l; and B, 0.34 mg/l. The microalgal preculture was inoculated to achieve an initial cell density of 0.15 × 10^8^ cells/ml. To maintain heterotrophic conditions, the culture flasks were wrapped in aluminum foil to exclude light. Cultivation was conducted on an orbital shaker at 120 rpm and 25°C for 6 days. pH and cell dry weight were measured, and molasses concentrations that resulted in relatively high biomass production were selected for further experimentation.

### Enhanced Heterotrophic Production through Response Surface Methodology (RSM)

Culture conditions were optimized using Response Surface Methodology (RSM) with a central composite design (CCD). Statistical analysis was conducted using Design-Expert software (version 7, Stat-Ease Inc., USA) to determine the optimal concentrations of AE and molasses for maximizing heterotrophic production of *Chlorella* sp. G049. The effects of AE and molasses concentrations were evaluated at three levels to assess their individual and interactive influences on three response variables: microalgal biomass production (g/l), lipid yield (g/l), and polyunsaturated fatty acid (PUFA) content (% of total fatty acid). A total of 13 experimental runs were performed based on the CCD, including triplicates at the central point to estimate experimental error. The interactions between the independent variables were analyzed using a second-order polynomial (quadratic) Eq. (1) as follows:

Y = *β_0_* + ∑*β_i_x_i_* + ∑*β_ii_x^2^_i_* + ∑*β_ij_x_i_x_j_* (1)

where *Y* is the predicted response, x_1_ and x_2_ represent the independent variables (AE and molasses concentrations, respectively), *β_0_* is the intercept term, *β_i_* represents the linear effects, *β_ii_* the quadratic effects, and *β_ij_* the interaction effects. Numerical optimization was performed using DesignExpert to determine the conditions that would maximize biomass production, lipid yield, and PUFA content. To validate the model, the experimental setup corresponding to the highest desirability score was carried out in triplicate. Three independent cultivation experiments were conducted under the predicted combination of AE and molasses concentration. The culture flasks were wrapped in aluminum foil to exclude light. Cultivation was conducted on an orbital shaker at 120 rpm and 25°C for 6 days. Results were reported as mean ± standard deviation, with a 95% confidence level.

### Analytical Methods

**Determination of biomass production.** Microalgal *Chlorella* sp. G049 biomass was estimated by cell counting using a hemocytometer. A strong linear correlation (R^2^ = 0.9981) was established between dry cell weight (DCW, g/l) and cell density (log 8 cells/ml), allowing for the conversion of cell counts to biomass using the following Eq. (2):

DCW = (3.0524 × Cell numbers) + 0.5054 (2)

**Determination of lipids.** Lipids were extracted from dried *Chlorella* sp. G049 biomass using a 2:1 (v/v) chloroform: methanol mixture, following the method described by Maneechote *et al*. [11]. The biomass–solvent mixture was subjected to sonication for 30 min, followed by centrifugation at 6,000 rpm for 15 min. The extraction was performed twice to ensure maximum lipid recovery. The combined supernatants were evaporated to remove the solvents, and the remaining lipid residue was dried in a hot air oven at 60°C before weighing. Lipid yield (LY, mg/l) was calculated using the following Eq. (3):

LY = [Lipid (mg) / DCW used (g)] × [ DCW from growth cultivation (g/l)] (3)

**Determination of Polyunsaturated Fatty Acids (PUFAs).** Fatty acid profiles of *Chlorella* sp. G049 were analyzed by converting extracted lipids into fatty acid methyl esters (FAMEs). A total of 10 mg of lipid extract was mixed with 1.5 ml of methanol, 0.5 ml of toluene, and 0.05 ml of 35% hydrochloric acid (HCl). The mixture was heated at 98°C for 1.5 h to complete the methylation reaction. After cooling, 1 ml of hexane was added, and the solution was thoroughly mixed. The FAMEs dissolved in the hexane phase were then analyzed using gas chromatography (Agilent GC 7890) equipped with an HP-5 column [12]. FAMEs were identified based on their retention times and verified using a standard mixture of FAME components. The yield of polyunsaturated fatty acids (PUFAs), expressed as a percentage of total fatty acids, was calculated using the following Eq. (4):

PUFA = [PUFA / (SFA + UFA)] × 100 (4)

where PUFA represents the weight percentage of polyunsaturated fatty acids, SFA is the percentage of saturated fatty acids, and UFA is the percentage of unsaturated fatty acids.

**Estimation of nutritional indices of lipids.** Several nutritional indices were calculated to assess the lipid profile of *Chlorella* sp. G049, including the ratio of polyunsaturated to saturated fatty acids (PS), the atherogenicity index (IA), the thrombogenicity index (IT), the ratio of hypocholesterolemic to hypercholesterolemic fatty acids (h/H), the health-promoting index (HPI), the unsaturation index (UI), the combined content of eicosapentaenoic acid and docosahexaenoic acid (SED), and the trans fatty acid (TFA) content, as described by Maneechote *et al*. [11].

**Estimation of biodiesel properties.** The FAME profiles of *Chlorella* sp. G049 lipids were analyzed to evaluate their biodiesel properties. These properties include saponification value (SV, mg KOH/g), iodine value (IV, g I_2_/ 100 g), cetane number (CN), degree of unsaturation (DU, % wt), long-chain saturation factor (LCSF, % wt), cold filter plugging point (CFPP, °C), high heating value (HHV, MJ/kg), cloud point (CP, °C), kinematic viscosity (v, in KV at 40°C in mm^2^/s), density (ρ, g/cm^3^), oxidative stability (OS, h), allylic position equivalents (APE), and bis-allylic position equivalents (BAPE). These characteristics were calculated based on the fatty acid profile of the lipids, as described by Pekkoh *et al*. [12].

**Determination of wastewater pollutants.** Chemical oxygen demand (COD) was analyzed according to standard methods outlined by the American Public Health Association [16]. The concentrations of nitrate (NO_3_^-^), phosphate (PO_4_^3-^), and ammonium (NH_4_^+^) were determined using a DR^2^100 spectrophotometer (HACH, USA).

### Statistical Analysis

All experimental results are presented as mean ± standard deviation (SD) from triplicate measurements. Statistical significance was determined using one-way analysis of variance (ANOVA), followed by Duncan’s Multiple Range Test (DMRT) at a confidence level of *p* < 0.05. All statistical analyses were conducted using SPSS Statistics software, version 17.0 (IBM Corp., USA).

## Results and Discussion

### Heterotrophic Cultivation of *Chlorella* sp. G049 Using Anaerobic Effluent

**Fig. 1:**
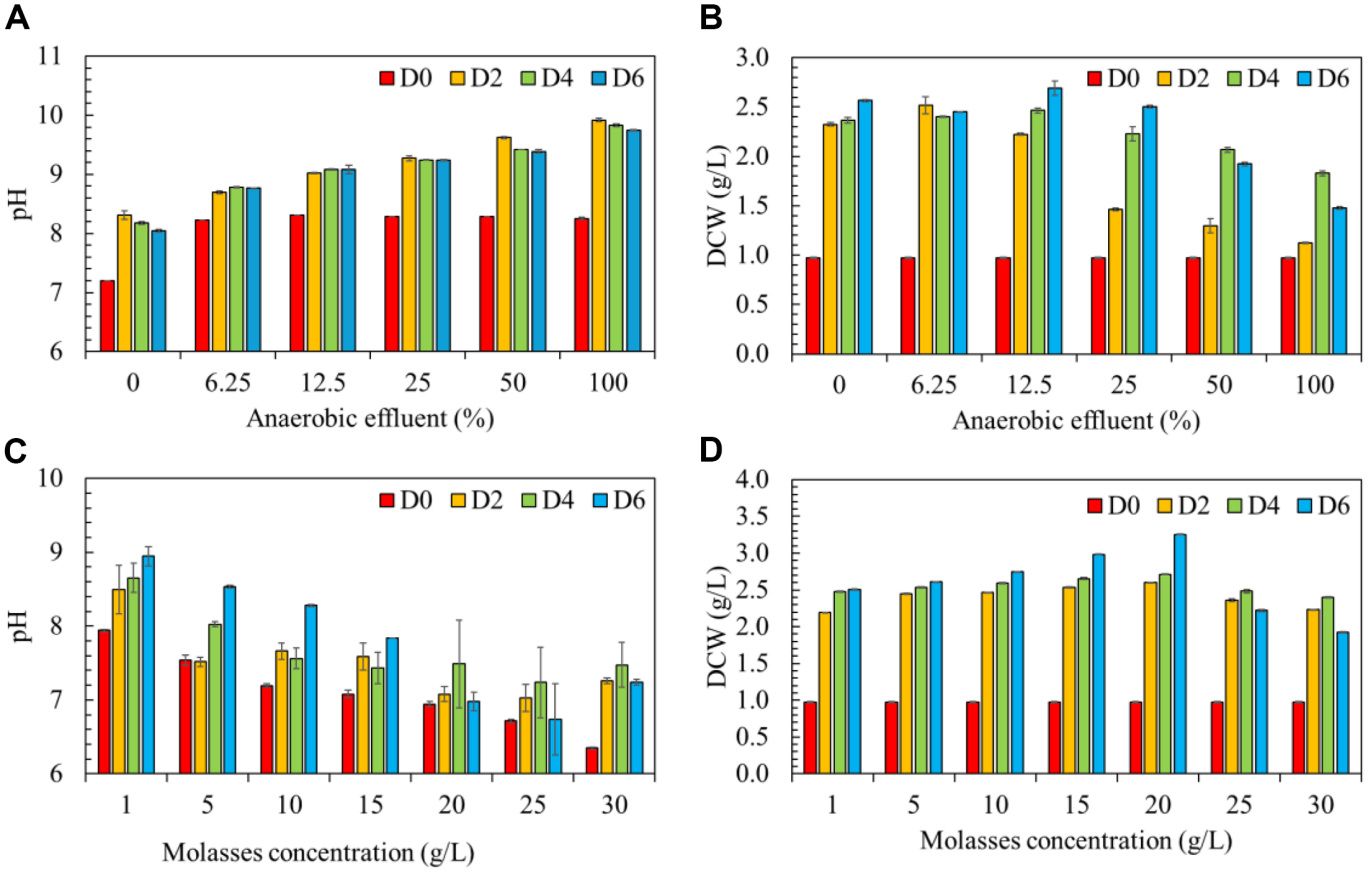
pH (A) and biomass production (B) of microalga *Chlorella* sp. G049 cultivated under different concentrations of anaerobic effluent (%); pH (C) and biomass production (D) of microalga *Chlorella* sp. G049 cultivated under different concentrations of molasses (g/l).

The effect of different anaerobic effluent (AE) dilution ratios (6.25%, 12.5%, 25%, 50%, and 100%) on the heterotrophic growth performance of *Chlorella* sp. G049 was evaluated and compared with a control culture grown in commercial JM medium. The initial pH of the AE ranged from 7.2 to 8.3, while the pH of the microalgal cultures during cultivation increased to between 8.03 and 9.74 (Fig. 1A). The variation in pH is attributable to the metabolic activity of microalgae, which alters the surrounding medium, thereby influencing their growth. The observed pH range (7–9) remained within the optimal limits for microalgal cultivation [11]. As illustrated in Fig. 1B, increasing AE concentrations up to 12.5% enhanced the biomass production of *Chlorella* sp. G049 under heterotrophic conditions. The highest biomass concentration recorded was 2.69 g/l at day 6 when 12.5% AE was used. In contrast, the control culture (0% AE, JM medium only) achieved a slightly lower biomass yield of 2.57 g/l. This improvement at low AE concentrations may be attributed to the nutrients derived from food waste present in the effluent, which enrich the culture medium with essential elements like nitrogen and phosphorus [7]. The organic matter content in the effluent, particularly in the form of chemical oxygen demand (COD), serves as an additional carbon source that supports heterotrophic growth [12]. Microalgae rapidly assimilate ammonium for amino acid biosynthesis and utilize phosphorus for nucleic acid and energy-related metabolic functions [7]. Therefore, low-to-moderate concentrations of AE can serve as a cost-effective alternative for nutrient supplementation in microalgal cultivation.

However, as AE concentration increased to 50% and 100%, biomass production declined significantly. At 50%and 100% AE, the biomass yields were 1.92 g/l and 1.48 g/l, respectively, indicating strong inhibition of microalgal growth. This decline may be due to excess nutrients, especially high COD and ammonium levels, which can become inhibitory or even toxic at high concentrations [17]. These findings align with previous studies. Kusmayadi *et al*. [18] reported that a 50% dilution of dairy wastewater yielded the highest biomass (3.2 g/l) and productivity (0.25 g/l/d) for *Chlorella sorokiniana* SU-1, while undiluted wastewater (100%) resulted in lower biomass (2.10 g/l) and productivity (0.16 g/l/d). In contrast, our study achieved comparable yields (2.69 g/l) at only 12.5% AE dilution, showing that effective biomass accumulation can be obtained at lower effluent concentrations. This is advantageous because it reduces the need for extensive nutrient supplementation, and minimizes inhibitory effects from high organic loads, and enhances process stability. In another related study, Chen *et al*. [17] demonstrated that optimal food waste filtrate concentration for microalgal cultivation was around 10%, with growth inhibition occurring at concentrations above 30%. *Cyanobacterium aponinum* exhibited complete growth suppression in 100% food waste filtrate, likely due to nutrient overload—especially ammonia toxicity. Rude *et al*.[7] also observed that *C. sorokiniana* grew best in 10% food waste permeate (FWP), achieving 0.274 g/l biomass, outperforming both 30% FWP (0.235 g/l) and the standard BG-11 medium (0.201 g/l). Our findings indicate that a lower AE concentration (12.5%) not only avoided inhibition but also maximized biomass yield. These findings indicate that our approach could be more cost-effective and scalable for wastewater bioremediation coupled with microalgal biomass production. Therefore, this study demonstrated that a 12.5% dilution of anaerobic effluent provided the most favorable conditions for the heterotrophic cultivation of *Chlorella* sp. G049. This condition supported optimal biomass accumulation by balancing nutrient availability and minimizing the inhibitory effects of excess organic load.

### Heterotrophic Cultivation of *Chlorella* sp. G049 Using Molasses

One of the primary limitations of using anaerobic effluent as a medium for microalgal cultivation is the low biomass yield. To enhance productivity, heterotrophic cultivation supplemented with an external carbon source such as molasses has been widely employed. Molasses is alkaline and rich in organic matter and nitrogen; however, it also contains a significant amount of inhibitor compounds that may impact algal growth [8]. In this study, the effects of different molasses concentrations (1, 5, 10, 15, 20, 25, and 30 g/l) on the heterotrophic growth of *Chlorella* sp. G049 using optimized AE (12.5%) were investigated over a 6-day cultivation period. The pH of the cultivation medium ranged from 6.9 to 8.9 (Fig. 1C), with the initial pH between 6.3 and 7.9—conditions generally favorable for microalgal growth. As shown in Fig. 1D, *Chlorella* sp. G049 grew well in molasses concentrations ranging from 1 to 25 g/l. The maximum biomass concentration (3.26 g/l) was achieved at 20 g/l of molasses. Molasses contains simple sugars such as glucose, sucrose, and fructose, which can serve as preferred carbon sources under heterotrophic conditions. In such environments, microalgae metabolize organic carbon through specialized pathways to produce ATP and biosynthetic precursors required for growth [8]. However, increasing the molasses concentration to 30 g/l resulted in a significant reduction in biomass (2.48 g/l). This decline is likely due to the inhibitory effects of excess nutrients, suspended colloids, and other recalcitrant compounds present in molasses, which may interfere with cellular metabolism, disrupt the Calvin cycle, and impair nutrient uptake. This phenomenon aligns with previous reports indicating that excessive sugar concentrations can inhibit microalgal growth due to substrate inhibition, a common limitation in batch cultures [19].

Although molasses supplied sufficient carbon to support heterotrophic growth, the relatively unchanged biomass yield observed (Fig. 1D) indicates that other factors might have limited further biomass accumulation. Nitrogen and phosphorus are key macronutrients required for protein and nucleic acid synthesis, and their insufficiency can restrict growth even under abundant carbon availability [19]. Similarly, the lack of certain trace elements (*e.g.*, iron, magnesium, zinc) may have constrained enzymatic activities critical for metabolic flux. In addition, molasses is a complex mixture that, apart from sugars, contains non-sugar organic compounds, minerals, and possible inhibitory substances such as phenolics [20, 21]. These components may have reduced the bioavailability of nutrients or imposed stress on the cells, thereby capping biomass productivity. This suggests that supplementation with balanced nutrients or pretreatment of molasses may be necessary to fully exploit its potential as a feedstock for high-density microalgal cultivation.

Similarly, Engin *et al*. [20] successfully cultivated *Micractinium* sp. ME05 in molasses-supplemented media, obtaining a maximum biomass of 3.73 g/l, highlighting the economic potential of molasses as a carbon source. Compared with previous studies, our findings demonstrate several distinct advantages. For instance, Yan *et al*. [21] reported that *Chlorella protothecoides* achieved optimal biomass at higher molasses concentrations (30–50 g/l), whereas our study found maximum biomass (3.26 g/l) at a significantly lower concentration (20 g/l). This lower requirement reduces substrate cost and risk of substrate inhibition, improving process efficiency. While Rahman *et al*. [22] observed that *Chlorella vulgaris* achieved the highest biomass (7.09 g/l) in normal molasses medium (1.0 g/l) after 8 days, with further increases in molasses concentration leading to decreased growth. In contrast, our study optimized biomass productivity (3.26 g/l) at a moderate molasses concentration (20 g/l) in just 6 days, reducing substrate costs and cultivation time. Additionally, Masoudi *et al*. [19] found the highest biomass (6.34 g/l) in 10% molasses wastewater without suspended solids, while suspended solids at higher concentrations significantly reduced growth. Our research found that an optimized approach with clarified molasses 20 (g/l) and AE (12.5%) supplementation minimized inhibitory effects and maintained higher biomass productivity. These differences highlight the greater process efficiency and potential of our system. Overall, the results from the present study indicate that molasses can serve as a cost-effective carbon source for enhancing microalgal growth under heterotrophic conditions. The optimal concentration for *Chlorella* sp. G049 was found to be 20 g/l, beyond which inhibitory effects limited biomass productivity. These findings underscore the importance of optimizing molasses concentration to balance nutrient supply and avoid substrate inhibition.

### Enhanced Heterotrophic Production through Response Surface Methodology (RSM)

This study differs from Sections 3.1 and 3.2, which investigated the effects of anaerobic effluent and molasses on biomass production alone using a one-factor-at-a-time (OFAT) approach. Those sections were designed to identify the preliminary concentration ranges at which *Chlorella* sp. G049 could grow effectively and accumulate biomass. Lipid and PUFA production were not assessed in those experiments because, under non-optimized conditions, imbalanced nutrient supply can suppress lipid biosynthesis and lead to inconsistent or misleading results regarding fatty acid profiles. In contrast, the present study utilized Response Surface Methodology (RSM), a multivariate statistical technique that offers several advantages over OFAT. Unlike OFAT, which evaluates one variable at a time and ignores potential interactions between factors, RSM simultaneously examines the effects and interactions of multiple variables, allowing for a more comprehensive understanding of the system [23]. This approach not only identifies the optimal concentrations of anaerobic effluent and molasses to enhance biomass production but also enables the simultaneous maximization of lipid yield and polyunsaturated fatty acid (PUFA) content under statistically optimized and physiologically balanced cultivation conditions.

**Statistical analysis.** The optimization of heterotrophic cultivation of *Chlorella* sp. G049 using anaerobic effluent (AE) and molasses was carried out using a central composite design (CCD) coupled with RSM (Table S1). The model equations were developed for three key responses: biomass concentration (Y_1_), lipid yield (Y_2_), and PUFA content (Y_3_). The polynomial regression equations were:

Y_1_ = 2.87 + 0.63A − 0.57B − 0.94AB + 0.041A^2^ − 0.40B^2^ (5)

Y_2_ = 66.21 + 24.39A + 16.85B + 9.75AB − 0.77A^2^ + 9.48B^2^ (6)

Y_3_ = 11.20 + 0.043A + 0.44B − 1.03AB + 0.48A^2^ − 0.97B^2^ (7)

According to Table 1, the analysis of variance (ANOVA) confirmed the significance of all three models at the 95% confidence level (*p* < 0.05), indicating that the models sufficiently explained the variability in the experimental data. The model F-values were 4.64, 4.62, and 4.74 for biomass, lipid yield, and PUFA, respectively, all indicating model significance. These results suggest that the models were appropriate for predicting the responses within the tested range. Adequate precision values for all models were above 6, indicating sufficient signal-to-noise ratios for model reliability. Furthermore, the non-significant lack-of-fit values (p > 0.05) for all responses support the adequacy of the models. Among the linear terms, anaerobic effluent (AE) concentration (factor A) had a statistically significant effect on biomass (*p* = 0.04) and lipid yield (*p* = 0.01), while molasses concentration (factor B) significantly influenced lipid yield (*p* = 0.04). The interaction term (AB) was significant for both biomass (*p* = 0.02) and PUFA content (*p* = 0.02), indicating that the combined effect of AE and molasses plays a crucial role in these responses.

**Fig. 2:**
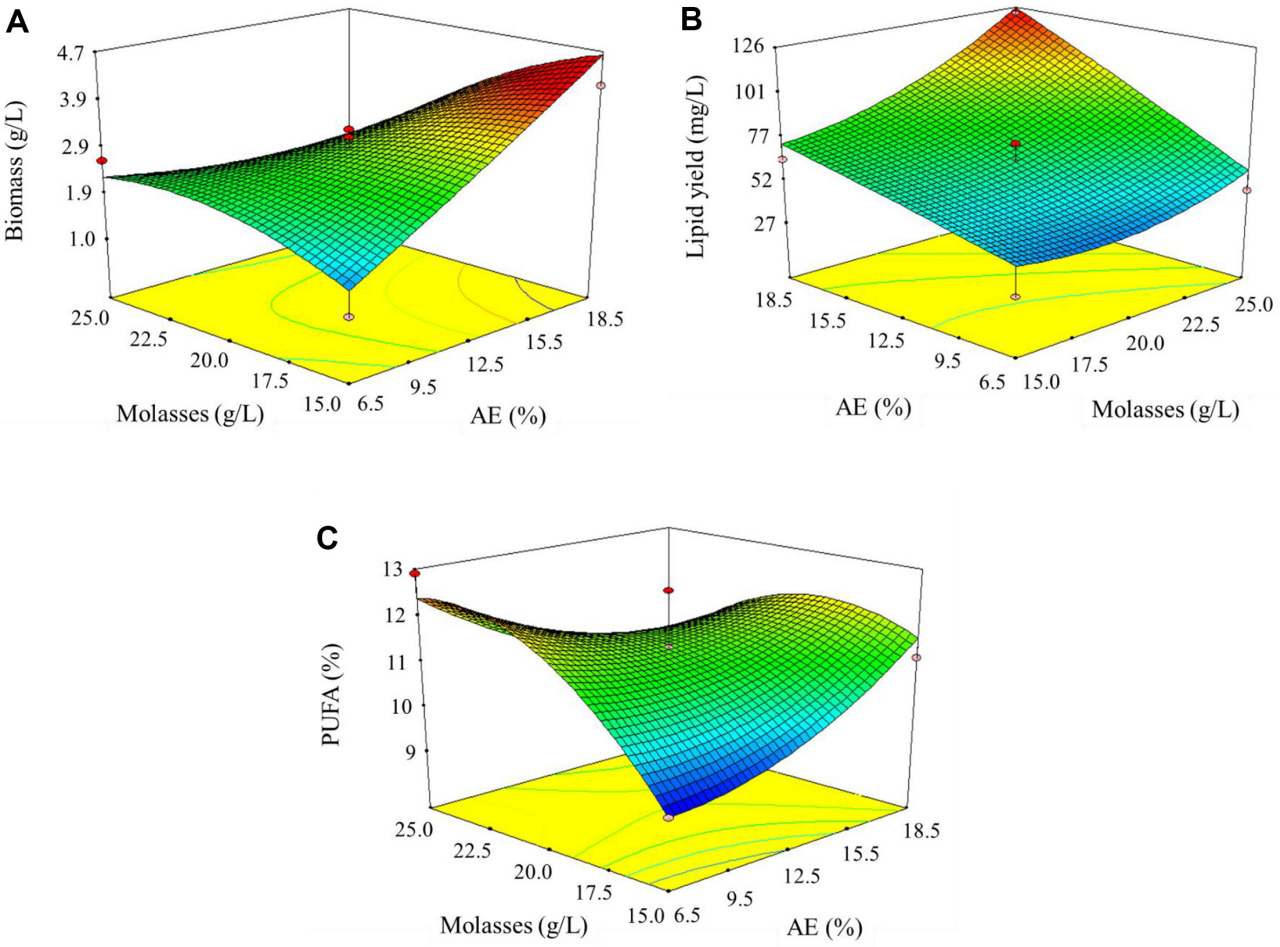
3D response surface plots of biomass production (A), lipid yield (B), and PUFA content (C) of *Chlorella* sp. G049.

**Effect on biomass production.** Biomass concentration ranged from 1.02 to 4.06 g/l across different combinations of AE and molasses concentrations (Table S1). The highest biomass production (4.06 g/l) was observed at 18.5%AE and 15 g/l molasses (Run 13), while the lowest was at 6.5% AE and 15 g/l molasses (Run 1). The model equation for biomass (Y_1_) suggests a positive linear influence of AE (coefficient = +0.63) and a negative effect from molasses (−0.57). However, the interaction between AE and molasses was significantly negative (−0.94), indicating that excessively high levels of both substrates together could hinder biomass accumulation. These results are consistent with the contour plots (Fig. 2A), where biomass peaked at moderate to high AE concentrations with moderate molasses supplementation. This suggests that the presence of organic carbon from molasses and nutrients from anaerobic effluent synergistically enhanced *Chlorella* heterotrophic growth, but only within optimal concentrations.

The AE likely supplied sufficient nitrogen and trace elements to support protein and nucleic acid synthesis, while molasses, a rich source of sucrose and glucose, fueled rapid cell proliferation under dark heterotrophic conditions. However, excessive carbon without balanced nitrogen may lead to carbon overflow metabolism, reducing growth due to osmotic stress [1]. It was anticipated that co-utilization of organic and inorganic nutrient sources would boost heterotrophic growth. This was validated, particularly the importance of moderate AE and molasses concentrations for maximum biomass. Similar biomass enhancements were reported by Hasnain *et al*.[24], who demonstrated improved algal biomass under optimal conditions using sugarcane molasses and supplemented nutrients. Moreover, Zhou *et al*. [25] noted that nutrient-rich waste streams significantly enhance biomass productivity when C:N ratios are optimized.

**Effect on lipid production.** Lipid yield exhibited a wide range, from 27 to 122 mg/l (Table S1). The highest lipid yield (122 mg/l) was recorded in Run 5 (18.5% AE and 25 g/l molasses), suggesting that both carbon and nitrogen-rich conditions favored lipid biosynthesis. The model (Eq. (6)) indicated strong positive coefficients for AE (+24.39), molasses (+16.85), and their interaction (+9.75), indicating that lipid accumulation was significantly enhanced by both substrates. While AE provided essential nutrients (particularly nitrogen and trace elements), molasses likely served as a readily assimilable carbon source, promoting acetyl-CoA generation required for fatty acid synthesis [25]. The response surface (Fig. 2B) clearly supports the synergistic effect, as lipid production increased with increasing levels of both factors. It is notable that while lipid accumulation typically occurs under nutrient-limited conditions, the presence of molasses may have induced lipid biosynthesis through carbon oversupply, even under moderate nitrogen availability from AE.

Lipid accumulation in microalgae typically increases under carbon-rich and nitrogen-limiting conditions [1]. However, in this study, AE likely provided enough nitrogen to support biomass growth without entirely suppressing lipid biosynthesis. The molasses, as a rapid carbon source, promoted acetyl-CoA flux towards fatty acid synthesis. It was anticipated that high carbon input would enhance lipid yield, especially under moderate nitrogen conditions. The results confirm this, indicating that AE concentrations above 18% and molasses around 25 g/l promote optimal lipid synthesis. These findings are in agreement with Jareonsin *et al*. [8], who reported enhanced lipid accumulation in *Chlorella* sp. AARL G015 under heterotrophic conditions with glucose supplementation. Rossi *et al*. [26] also demonstrated that lipid content is maximized when microalgae are cultivated with a controlled C:N ratio using wastewater and sugar-rich media.

**Effect on PUFA content.** PUFA content in the biomass varied from 9.19% to 12.61%. The highest PUFA percentage was obtained in Run 11 (6.5% AE and 25 g/l molasses), suggesting that a lower AE concentration combined with high molasses favored unsaturated fatty acid biosynthesis. The regression model (Eq. (7)) showed that while the individual effects of AE and molasses were relatively small (coefficients +0.043 and +0.44, respectively), the interaction term (−1.03) and the quadratic term for molasses (−0.97) were significant, indicating that excessive levels could suppress PUFA accumulation. This observation could be attributed to the potential imbalance between carbon and nitrogen ratios affecting desaturase activity or membrane lipid remodeling under different nutrient regimes [1]. As seen in Fig. 2C, PUFA levels peaked at moderate molasses with low AE, supporting a strategy where carbon abundance and controlled nitrogen levels drive PUFA biosynthesis.

PUFA biosynthesis is sensitive to nutrient stress and environmental conditions [4]. While high carbon levels from molasses supported overall lipid synthesis, excessive molasses likely triggered metabolic imbalances that reduced desaturation activity [24], as reflected in the significant negative B^2^ coefficient (−0.97). The lack of a significant AE effect suggests that nitrogen supply did not directly modulate desaturation enzymes. It was anticipated that heterotrophic conditions may limit PUFA accumulation due to reduced oxygen availability and lower desaturase activity [27]. However, the results show that *Chlorella* sp. G049 maintains substantial PUFA levels under these conditions, likely due to strain-specific metabolic pathways. Sun *et al*. [27] reported that PUFAs can still accumulate under heterotrophic or mixotrophic conditions, especially when oxygen transfer or desaturation steps are not limiting. The current findings align with these studies, suggesting that carefully balanced carbon input (15–25 g/l molasses) enhances PUFA retention in lipids.

**Numerical optimization and verification of the model.** To evaluate the predictive reliability of the developed RSM model, numerical optimization was performed using a desirability function approach. This approach aimed to identify the optimal combination of AE and molasses concentration that would simultaneously maximize biomass concentration, lipid yield, and PUFA content under heterotrophic cultivation of *Chlorella* sp. G049. The optimal conditions determined through RSM were 18.5% AE and 18.18 g/l molasses, yielding a composite desirability score of 0.758. This value, which approaches the ideal value of 1.0, indicates a high level of agreement between the model's prediction and the experimental goals. The relatively high desirability score of 0.758 obtained in this study compares favorably with other RSM-optimized studies, where typical desirability values range between 0.70 and 0.85 for multi-response optimizations. For instance, Zakir Hossain *et al*. [28] and Rahmati *et al*.[29] reported a desirability score of 0.66-1.00 in their RSM-based optimization of microalgal biomass and lipid productivities from *Chlorella kessleri* and *C. vulgaris* using various nutrient source.

**Fig. 3:**
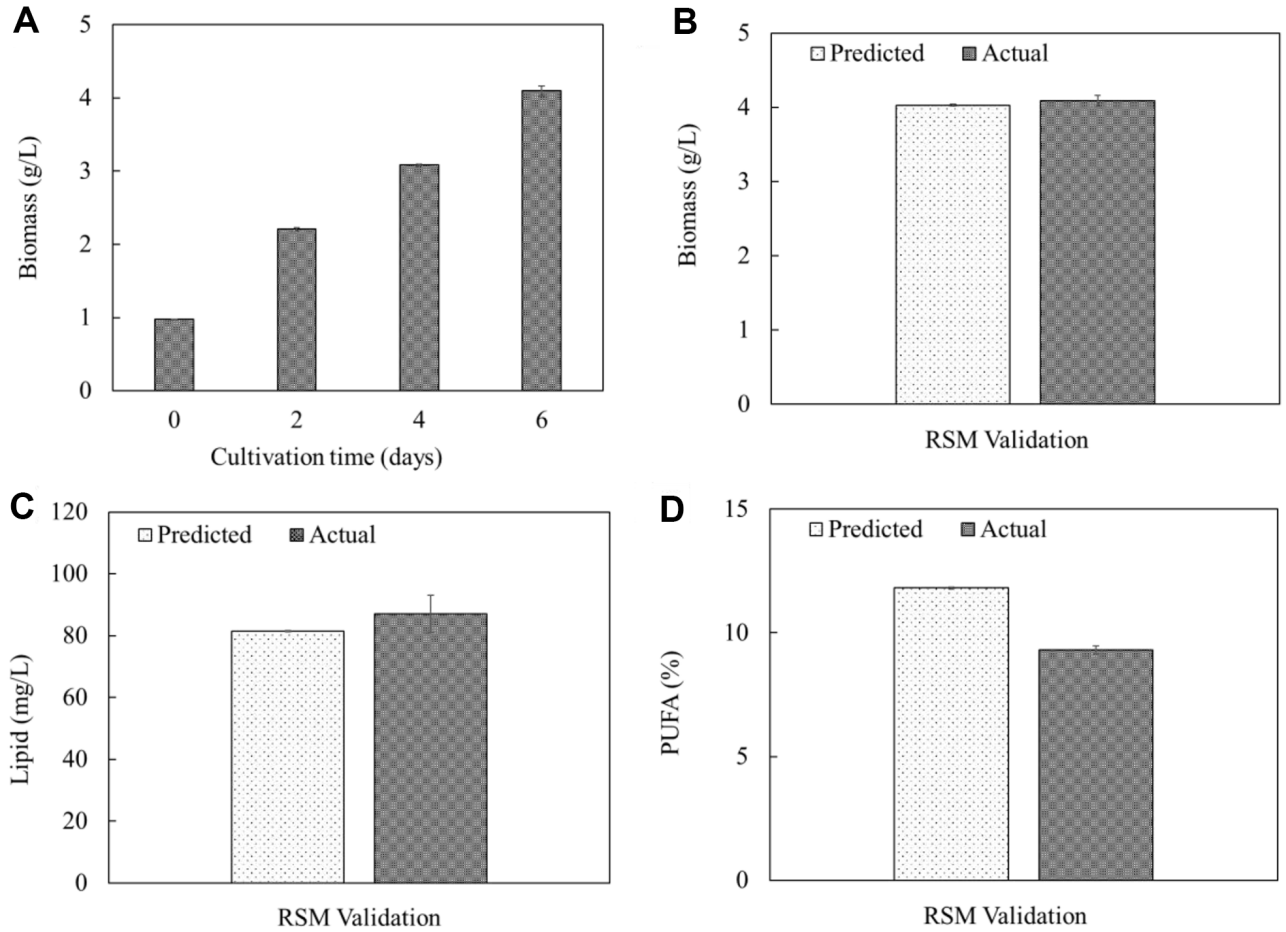
Time-course profile of biomass accumulation (A) RSM validation results comparing predicted and actual values of biomass production (B) lipid content (C) and PUFA composition (D). All experiments were performed in triplicate (*n* = 3), and each measurement was conducted in three technical replicates. Data are presented as mean ± standard deviation (SD), with error bars representing SD.

To validate the accuracy of the model, three independent cultivation experiments were conducted under the predicted combination of AE (18.5%) and molasses concentration (18.18 g/l). The RSM model forecasted a biomass yield of 4.03 g/l, a lipid yield of 81.43 mg/l, and a PUFA content of 11.88% (Table S1 and Fig. 3). Experimentally, the observed biomass concentration was slightly higher at 4.09 g/l, and the lipid yield reached 87.00 mg/l, which exceeded the predicted value by approximately 6.86%. In contrast, the PUFA content was somewhat lower than expected, with an observed value of 9.30%, deviating from the prediction by approximately 22.9%. The strong correlation between predicted and experimental values for biomass and lipid yield confirms the model’s robustness and predictive power. These results validate the hypothesis that an optimized combination of AE and molasses can significantly enhance biomass productivity and lipid accumulation under heterotrophic conditions. The use of AE as a nitrogen and micronutrient source, coupled with molasses as a carbon-rich substrate, provides a balanced C:N ratio that supports cell growth while driving lipid biosynthesis. The slightly higher experimental values for biomass and lipid yield may reflect unaccounted synergistic effects in the actual cultivation environment, such as better nutrient bioavailability and interactions that favored algal metabolism.

The larger deviation observed in PUFA content prediction, however, highlights the limitations of the model in accurately capturing complex biochemical responses, particularly those influenced by intracellular regulation and environmental microconditions. PUFA biosynthesis in microalgae is known to be sensitive to oxygen availability, redox state, and specific enzymatic activities (*e.g.*, desaturases) [24, 27], which may not be fully accounted for by the AE and molasses variables alone. Additionally, the inverse relationship between total lipid content and PUFA proportion may have contributed to this discrepancy, as rapid accumulation of neutral lipids can dilute the relative concentration of PUFAs [27]. Similar observations have been reported in previous studies, such as Adesanya *et al*. [30] and Kumar *et al*. [31], where optimization models accurately predicted biomass and lipid trends but showed less precision for fatty acid composition due to the complexity of metabolic regulation. The deviation in PUFA prediction highlights the need for further refinement, possibly through inclusion of oxygenation parameters or use of enhanced metabolic models (*e.g.*, flux balance analysis) in future optimization.

Overall, the numerical optimization and model verification highlight the effectiveness of RSM as a tool for designing multi-objective cultivation strategies for microalgal systems. The high desirability score and close agreement between predicted and experimental values reinforce the feasibility of using waste-derived resources such as anaerobic effluent and molasses for cost-effective and sustainable microalgal production. Although PUFA content showed some deviation, the overall high desirability score confirms that the model is effective in guiding multi-objective optimization. These findings contribute to the development of economically viable and environmentally sustainable algal bioprocesses using waste-derived feedstocks.

### Microalgal Performance for Use as Nutritional Lipids

Nutritional lipids are fundamental components of a balanced diet, playing critical roles in human and animal physiology. These include energy storage, maintenance of cell membrane integrity, and the synthesis of hormones. Polyunsaturated fatty acids (PUFAs), in particular, are known to confer numerous health benefits, such as promoting cardiovascular health, exerting anti-inflammatory and immunomodulatory effects, and providing antiviral, antimicrobial, neuroprotective, and anthelmintic activities [4]. Moreover, PUFAs are involved in the biosynthesis of prostaglandins and thromboxanes, compounds that help regulate cholesterol and triglyceride levels and prevent inflammatory-related disorders, including arthritis and skin diseases [9]. The potential of PUFAs extends beyond human health, as they are also beneficial for animal health and can serve as high-quality feed resources for feed applications [4]. Fatty acid profiling of lipids from *Chlorella* sp. *G049* was cultivated under optimal RSM conditions as shown in Table 2. The PUFA value (9.30%) of this strain aligns with values observed in other algae strains [4, 9, 12]. In addition, the amount of PUFA depends on the algae species and the cultivation conditions.

To evaluate the nutritional and functional quality of lipids from *Chlorella* sp. G049, several indices are used: polyunsaturated fatty acids to saturated fatty acid (PUFA/SFA) ratio, atherogenic index (AI), thrombogenic index (TI), hypocholesterolemic to hypercholesterolemic (HH) ratio, health-promoting index (HPI), and unsaturation index (UI). These indices offer comprehensive insight into the potential health impacts of dietary lipids, as shown in Table S2, and are compared to lipid sources from animals, plants, marine organisms, and other microalgae [4, 11, 13, 32].

The PUFA/SFA ratio is a key indicator of lipid quality, especially in the context of cardiovascular health. PUFAs are known to reduce LDL cholesterol and total serum cholesterol, whereas SFAs, particularly C10:0 to C20:0, are associated with increased cholesterol levels. A higher PUFA/SFA ratio suggests a healthier lipid profile. For *Chlorella* sp. G049, this ratio was 0.14, aligning with values observed in red seaweed (0.14–2.12), cattle (0.11–0.20), and lamb (0.13–0.37), but lower than those found in *Spirulina* sp., sunflower oil, and commercial plant protein-based sources (1.01–4.94) (Table S2). According to Chen and Liu [32], typical PUFA/SFA ratios across food groups include seaweed (0.42–2.12), fish (0.50–1.62), and shellfish (0.20–2.10). While the ratio is modest, our cultivation approach did not involve genetic modification or enrichment strategies, suggesting that a nutritionally comparable profile can be obtained using a simple, cost-effective approach and may still provide beneficial effects when integrated into a balanced diet.

The AI estimates the potential of fatty acids to contribute to the formation of atheromatous plaques in arteries. It is calculated based on the concentration of pro-atherogenic SFAs relative to UFAs. Lower AI values are desirable and suggest a reduced risk of atherosclerosis. The AI value (2.62) of *Chlorella* sp. G049 aligns with values observed in dairy products (1.42–5.13) and seaweeds (0.38–2.87), indicating a reduced risk for atherosclerosis (Table S2). Importantly, this value was achieved without lipid refining, highlighting the strain’s natural potential to produce heart-healthy lipids.

TI assesses the likelihood of fatty acids to promote thrombus (blood clot) formation. It balances pro-thrombogenic SFAs (*e.g.*, C12:0, C14:0, C16:0) against anti-thrombogenic MUFAs and PUFAs [32]. *Chlorella* sp. G049 lipid displayed a TI value of 3.52. These are higher than the optimal range, as lower TI values indicate better cardiovascular safety. For comparison, TI values for seaweeds span 0.46–5.75, while values for fish (0.06–0.72) and crops (0.14–0.18) are notably lower. These results suggest that while *Chlorella* lipid offers health benefits, its TI might be improved through selective strain enhancement or lipid refining.

HPI is the inverse of the AI and is used to evaluate the overall beneficial impact of dietary lipids. Higher HPI values denote a healthier lipid profile. The HH ratio measures the balance between fatty acids that promote a decrease in cholesterol (hypocholesterolemic, *e.g.*, oleic and linoleic acid) and those that raise it (hypercholesterolemic, primarily SFAs). Our HPI value (0.38) is comparable to *Rhopalodia* sp. (1.06) and dairy products (0.16–0.68)(Table S2), while the HH ratio (0.41) is similar to dairy products (0.32–1.29). This suggests *Chlorella* sp. AARLG049 could serve as a competitive alternative to traditional sources of functional lipids.

The UI reflects the total degree of unsaturation in a lipid profile and correlates with the oxidative stability and health-promoting potential of the lipids. It is calculated by multiplying the percentage of each unsaturated fatty acid by the number of double bonds it contains. *Chlorella* sp. G049 lipid had a UI of 43.58, lower than dairy products (86–120) and other algae (50.63–257.07) (Table S2). Though not optimal, these values suggest *Chlorella* lipid is a viable source of unsaturated fatty acids. However, the UI does not distinguish between n-3 and n-6 PUFAs, which have different physiological roles. n-3 PUFAs, such as EPA and DHA, are especially valued for their anti-inflammatory and neuroprotective properties [32]. Compared with previously reported animal, plant, and algal lipid sources (Table S2), *Chlorella* sp. AARLG049 demonstrates competitive nutritional indices—particularly a moderate AI and a favorable HPI—while being cultivated under environmentally sustainable conditions. Unlike fish oil and animal fats, *Chlorella* lipids can be produced at scale with consistent composition and without dependency on fluctuating natural stocks. These advantages make it a strong candidate for functional food and nutraceutical applications.

Although AE and molasses can effectively supplement nutrients in microalgal cultivation, products intended for human consumption require additional processing to ensure safety. In contrast, AE- and molasses-based cultivation is more practical and suitable for feed purposes, where safety standards are less restrictive and the focus is on cost-effective biomass production. Furthermore, this approach supports sustainability and the circular bioeconomy by converting low-value byproducts into valuable microalgal biomass, reducing waste and promoting resource efficiency.

### Microalgal Performance for Use as Biodiesel Feedstock

The fatty acid composition of lipids extracted from *Chlorella* sp. G049 was analyzed following optimization, and the resulting biodiesel properties were evaluated. Microalgal lipids can be converted into fatty acid methyl esters (FAMEs) via transesterification. Since FAMEs are the primary constituents of biodiesel, their composition significantly influences biodiesel quality [4]. It is important to note that fatty acid profiles vary among microalgal species, depending on growth stages and cultivation conditions [1]. In this study, the effects of anaerobic effluent and molasses supplementation on the fatty acid profile of *Chlorella* sp. G049 under optimized heterotrophic conditions were investigated. As shown in Table S3, this strain predominantly produced fatty acids with 16 to 18 carbon atoms, a desirable range for high-quality biodiesel production. Under optimal conditions (18.50%anaerobic effluent and 18.18 g/l molasses), *Chlorella* sp. G049 exhibited a high proportion of C16–C18 fatty acids, comprising 82.49% of total fatty acids. Saturated fatty acids (SFAs) accounted for 64.36%, while unsaturated fatty acids (UFAs) made up 35.64%, with monounsaturated fatty acids (MUFAs) and polyunsaturated fatty acids (PUFAs) comprising 26.33% and 9.30%, respectively. Palmitic acid was the dominant fatty acid, followed by oleic, linoleic, and linolenic acids. The optimized conditions increased palmitic acid content, elevating the SFA/UFA ratio. This shift is beneficial, as higher SFA content enhances biodiesel properties such as cetane number, oxidative stability, and thermal resistance [11]. Recent studies have demonstrated that wastewater-grown microalgae can yield fatty acid profiles suitable for biodiesel, offering a cost-effective alternative to synthetic media [11]. For example, *Chlorella* sp. AARL G015 cultivated heterotrophically showed over 90% of C16–C18 fatty acids, with UFA and PUFA contents reaching 59% and 36%, respectively [8]. By comparison, *Chlorella* sp. G049 in this study achieved 78.2% C16–C18 fatty acids with a higher SFA content (68.8%), which is advantageous for biodiesel stability and cetane number, even though PUFA levels were lower. This balance improves oxidative stability and fuel quality without requiring additional refining. Similarly, *C. sorokiniana* EAKI grown in untreated dairy wastewater had C16–C18 contents between 63.75% and 73.83%, SFAs between 38.84% and 44.88%, and UFAs between 22% and 33% [33]. Supporting these findings, Wang *et al*. [34] reported that *Chlorella pyrenoidosa* grown in municipal wastewater exhibited high unsaturation (92.1% C16–C18) and SFA contents (40.8%), indicating their suitability for biodiesel production. A comparison of fatty acid profiles under optimal and alternative conditions is provided in Table S3.

The potential biodiesel fuel characteristics of microalgal lipids were assessed following optimization under heterotrophic conditions (Table S4). Those include saponification value (SV), cetane number (CN), iodine value (IV), degree of unsaturation (DU), cold filter plugging point (CFPP), long chain saturation factor (LCSF), higher heating value (HHV), oxidative stability (OS), kinematic viscosity (ν), and density (ρ). This research assesses the biodiesel characteristics of lipids from *Chlorella* sp. G049 and compares them with three major international standards: the European biodiesel standard (EN14214), the American Society for Testing and Materials (ASTM D6751), and Thailand’s National Standards (TH2020). Table S4 summarizes the fuel characteristics of biodiesel produced from *Chlorella* sp. G049 lipids, highlighting its adherence to major international standards and its similarity to biodiesel obtained from other algal sources.

The saponification value (SV) represents the milligrams of KOH required to saponify 1 gram of oil. It reflects the average molecular weight and the length of the carbon chain in the microalgal fatty acids. A higher saponification value (SV) signifies a greater proportion of short-chain fatty acids in the microalgal lipids [4]. The SV of *Chlorella* sp. G049 was 211.64 mg KOH/g oil. These values are within the range reported for other microalgae (188.1–208.2 mg KOH/g oil). The iodine value (IV), which reflects the level of unsaturation and oxidative stability [9], was measured at 40.64 g I_2_/100g for *Chlorella* sp. G049. This complies with the European biodiesel standard (EN14214) limit of less than 120 g I_2_/100g and is consistent with values reported for other algae (55.07–91.10 g I_2_/100g). These values indicate a low level of unsaturation and a high stability of the biodiesel to oxidation. The cetane number (CN) reflects the ignition quality of a liquid fuel and tends to increase with higher saturated fatty acid (SFA) content [10]. The CN for *Chlorella* sp. G049 was as high as 62.95. These values also meet the minimum requirements of both ASTM D6750 (≥47) EN14214, and TH 2020 (≥51) standards, aligning well with CN values observed in other algae (48.2–60.02) and confirming the good ignition quality of microalgae-derived biodiesel.

The degree of unsaturation (DU) for biodiesel derived from *Chlorella* sp. G049 was 44.94, which is close to the range observed in other algal species (66–98). A lower DU indicates a higher cetane number and improved oxidative stability of the liquid fuel [12]. The cold filter plugging point (CFPP), which indicates the minimum temperature at which biodiesel can operate effectively [10], was measured at 24.37°C for *Chlorella* sp. G049, within the range reported for other algal biodiesels (-1.87°C to 67.45°C). The long-chain saturation factor (LCSF) for *Chlorella* sp. G049 was recorded at 13.00°C, which is consistent within the range reported for other algal species (4.65–26.7°C). The estimated higher heating value (HHV) of biodiesel produced from microalgae under these conditions was 40.14, which is similar to the HHV range of plant-based biodiesel (38–41) [35]. These values are within the range observed in other microalgae (HHV: 32.5–40 MJ/kg).

*Chlorella* sp. G049 also exhibited an oxidation stability (OS) of 15.27 h, meeting the requirements of ASTM D6750, EN 14214, and TH 2020 standards (>3 h). The OS of other algae values was 6.2-8.4 h. The cloud point (CP) refers to the temperature at which a liquid starts to appear cloudy as solid particles or crystals begin to form [12]. For *Chlorella* sp. G049, the CP was measured at 13.59°C, while for other microalgae it ranged from -4.0 to 12.38°C. The kinematic viscosity (ν), which affects both fuel flow and combustion [35], was recorded at 6.72 mm^2^/s for *Chlorella* sp. G049. This value is near the ASTM D6750 standard range of 1.9–6.0 mm^2^/s and consistent with the range observed in other microalgae (2.6–4.8 mm^2^/s). The density (ρ), which play a critical role in energy content and combustion efficiency [10], was measured at 0.98 g/cm^3^. While this slightly exceeds the limits set by EN14214 (0.86–0.90 g/cm^3^) and ASTM D6750 (0.85–0.90 g/cm^3^), it is still within the observed range reported for other algal species (0.87–6.84 g/cm^3^).

Overall, when compared with previous studies on microalgae-based biodiesel (Table S3), *Chlorella* sp. G049 demonstrated several advantages. It produced a high proportion of C16–C18 fatty acids (82%), comparable or superior to other *Chlorella* and *Scenedesmus* strains, while maintaining a high cetane number (62.95) and excellent oxidation stability (15.27 h) (Table S4). These properties ensure better engine performance, longer storage stability, and compliance with major international standards (ASTM D6750, EN 14214, and TH2020 standards) without requiring extensive post-processing. Additionally, the *Chlorella* sp. G049 strain's ability to grow heterotrophically in anaerobic effluent supplemented with molasses can serve as a suitable feedstock for biodiesel production and reduce production costs and environmental impact and support a circular bioeconomy.

### Microalgal Performance for Wastewater Treatment

In addition to high-value biomass and lipid production, *Chlorella* sp. G049 exhibited excellent potential for wastewater remediation under optimized heterotrophic conditions (18.5% anaerobic effluent and 18.19 g/l molasses (COD: 3,432 mg/l, N–NO_3_: 7.5 mg/l, N–NH_4_: 52.30 mg/l, P–PO_4_: 12.5 mg/l)). After six days of cultivation, the system achieved significant pollutant reductions: chemical oxygen demand (COD) was reduced by 91.79%, ammonium (NH_4_^+^) by 63.19%, nitrate (NO_3_^-^) by 73.21%, phosphate (PO_4_^3-^) by 96.76%, and sugar consumption by 57.07% (Table S5). These removal efficiencies demonstrate the strain’s capacity to serve as a dual-purpose platform for both resource recovery and effluent polishing in circular bioeconomy systems.

The COD reduction indicates effective assimilation and oxidation of organic substrates present in the medium. Compared to similar heterotrophic systems, the 91.79% COD removal observed in this study is notably higher than the 89.1% reported by Kusmayadi *et al*. [18] for *Chlorella sorokiniana* in diluted dairy wastewater and also exceeds the 61–89% range reported by other effluent types (Table S5). This improved performance is attributable to the optimized C:N ratio and the use of molasses-derived bioavailable sugars, which likely enhanced heterotrophic metabolism and organic substrate assimilation.

The microalga also achieved notable nitrogen removal, assimilating both ammonium and nitrate. Nitrogen metabolism in microalgae is typically dominated by ammonium uptake, as it is energetically favorable compared to nitrate assimilation, which requires reduction to ammonium via nitrate and nitrite reductases [4]. In this study, the relatively high ammonium removal (63.19%) suggests that *Chlorella* sp. G049 possesses an efficient ammonium assimilation pathway, likely involving active uptake through AMT-type transporters and rapid conversion into glutamine and glutamate via the GS-GOGAT cycle [9]. Nitrate removal (73.21%) was also significant, indicating that the strain can maintain nitrate reductase activity under heterotrophic conditions—an uncommon feature among many algal strains, which typically downregulate this pathway in the absence of light [4].

Phosphate removal reached 96.76%, surpassing values reported in earlier studies using both photoautotrophic and mixotrophic systems. For instance, Rahman *et al*. [22] reported 80.8% phosphate removal in *Chlorella vulgaris* cultivated in molasses-enriched media, while Jareonsin *et al*. [8] observed 76% removal using *Chlorella sorokiniana* AARL G015 in 39.45% (v/v) of the poultry wastewater with 57.03 ml/l of molasses. The superior phosphate uptake observed here can be attributed to high bioavailability in the anaerobic effluent and possibly the presence of inducible high-affinity phosphate transporters activated under phosphate-limiting intracellular conditions.

Although COD removal reached 91.79%, sugar consumption from molasses was only 57%, and biomass productivity did not fully reflect the available carbon source (Table S5). This discrepancy can be attributed to the complex composition of molasses and AE. COD represents total oxidizable organic matter, including sugars, proteins, amino acids, organic acids, lipids, and colored compounds such as melanoidins. Under heterotrophic conditions, *Chlorella* can metabolize multiple carbon sources beyond fermentable sugars, and partial oxidation of organics for respiration further reduces COD without corresponding sugar uptake. Additionally, analytical sugar quantification detects only specific fermentable carbohydrates, which underestimates total carbon utilization. Molasses contains not only fermentable sugars (sucrose, glucose, fructose) but also non-fermentable components and inhibitory substances (*e.g.*, phenolics, ash, heavy metals), which may limit microbial utilization [19-22]. Likewise, AE provided residual COD, ammonium, phosphate, and micronutrients, but may also contain inhibitory compounds such as volatile fatty acids or residual antibiotics [7, 12, 17]. These factors likely contributed to the incomplete sugar consumption and limited biomass yield despite the high COD removal. Pretreatment of molasses and AE, or nutrient supplementation, may therefore be necessary to enhance conversion efficiency and improve algal productivity.

From a regulatory perspective, the pollutant removal levels achieved in this study are sufficient to meet or exceed discharge standards set by many national and international environmental agencies [36]. For example, Thailand’s Ministry of Natural Resources and Environment stipulates effluent standards of <120 mg/l COD, <15 mg/l total nitrogen, and <1 mg/l total phosphorus for wastewater discharge into public water bodies [37]. The treated effluent from this microalgal system would comply with these criteria, demonstrating its potential for pre-discharge polishing or as part of decentralized wastewater treatment in agro-industrial zones.

Moreover, the integration of microalgal bioremediation into existing anaerobic digestion infrastructures could transform waste liabilities into bioresource assets. The nutrient-rich biomass can be valorized further into biofertilizers, animal feed, or high-value lipid products [1], while the clarified effluent could be reused for irrigation or safely discharged. This approach aligns with SDG 6 (clean water and sanitation) and SDG 12 (responsible consumption and production) [38], supporting regional and global environmental policy objectives.

Overall, the wastewater treatment performance of *Chlorella* sp. G049 compares favorably with previously reported microalgal systems (Table S5). This strain demonstrated higher COD and phosphate removal efficiencies and competitive nitrogen removal rates. The observed performance compares favorably with literature benchmarks and aligns with regulatory discharge limits. Additionally, the system generates nutrient-rich biomass that can be valorized into biofertilizers, animal feed, or lipid-based bioproducts, further supporting its integration into circular bioeconomy models.

### Future Perspectives

In this study, heterotrophic cultivation was conducted at the flask scale, which inherently limits oxygen transfer and aeration. Such limitations can restrict carbon and nutrient utilization, thereby constraining biomass productivity. For scale-up production, the use of fermenters is recommended, as they allow precise control of aeration, mixing, pH, and nutrient supply, supporting more efficient heterotrophic growth. Employing controlled bioreactors would not only enhance biomass yield but also improve the reproducibility and consistency of nutrient conversion, making the process more suitable for industrial applications. Furthermore, higher efficiency in nutrient utilization and biomass production contributes to sustainability and cost-effectiveness by maximizing output while minimizing resource input and waste generation, aligning with circular bioeconomy principles.

## Conclusion

This study successfully optimized the heterotrophic cultivation of *Chlorella* sp. G049 using anaerobic effluent and molasses, resulting in significant enhancements in both biomass and lipid yields. The extracted lipids demonstrated dual functionality—nutritional viability and compliance with international biodiesel standards—thereby supporting their application in the food, feed, and energy sectors. Simultaneously, the cultivation system exhibited high efficiency in wastewater treatment, achieving substantial removal of key pollutants including COD, ammonia, nitrate, and phosphate. These outcomes highlight the feasibility of valorizing industrial and agricultural waste streams through a sustainable, zero-waste microalgal bioprocess. The findings contribute meaningfully to circular bioeconomy strategies, offering a scalable and environmentally responsible approach for producing value-added algal bioproducts while addressing environmental concerns. Future investigations should focus on pilot-scale validation, metabolic engineering to enhance PUFA biosynthesis, and comprehensive techno-economic analyses to support industrial-scale implementation.

## Supplemental Materials

Supplementary data for this paper are available on-line only at http://jmb.or.kr.



## Figures and Tables

**Table 1:** Model coefficients and analysis of response variance estimated by ANOVA.

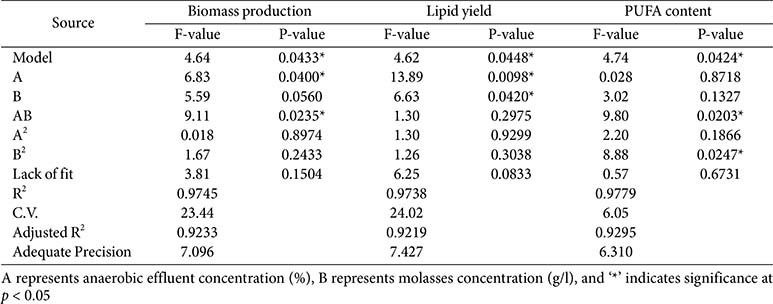

**Table 2:** Fatty acid profile of lipids extracted from *Chlorella* sp. G049 cultivated under optimal RSM conditions.