# Supplementary material for: Sustainable PUFA-Rich Lipid-Accumulating Biomass Production via Dual Waste Valorization Using Heterotrophic Microalgae Cultivated on Anaerobic Effluent and Molasses
Source: J Microbiol Biotechnol. 2025 Oct 28;35:e2506037. doi: 10.4014/jmb.2506.06037 (PMC12603369; doi:10.4014/jmb.2506.06037)
Supplement: Supplementary file 1 [file jmb-35-e2506037-supple.pdf]

J. Microbiol. Biotechnol. 2025. 35: e2506037
https://doi.org/10.4014/jmb.2506.06037

Supplementary Tables

Sustainable PUFA-Rich Lipid-Accumulating Biomass Production via Dual Waste Valorization Using Heterotrophic Microalgae Cultivated on Anaerobic Effluent and Molasses

Wageeporn Maneechote [1,2,3], Wasu Pathom-aree [2,4], Apiwit Kamngoen [1], Antira Wichaphian [1], Benjamas Cheirsilp [5], Kuan Shiong Khoo [6], Shuhao Huo [7], Piroonporn Srimongkol [8] and Sirasit Srinuanpan [1,2,3,4,8]*

[1] Microbial Biorefinery and Biochemical Process Engineering Research Group, Chiang Mai University, Chiang Mai 50200, Thailand
[2] Department of Biology, Faculty of Science, Chiang Mai University, Chiang Mai 50200, Thailand
[3] Office of Research Administration, Office of the University, Chiang Mai University, Chiang Mai 50200, Thailand
[4] Center of Excellence in Microbial Diversity and Sustainable Utilization, Faculty of Science, Chiang Mai University, Chiang Mai 50200, Thailand
[5] Center of Excellence in Innovative Biotechnology for Sustainable Utilization of Bioresources, Faculty of Agro-Industry, Prince of Songkla University, Hat Yai, Songkhla, 90110, Thailand
[6] Department of Chemical Engineering and Materials Science, Yuan Ze University, Taoyuan, Taiwan
[7] School of Food and Biological Engineering, Jiangsu University, Zhenjiang 212013, China
[8] High-Value Food from Mushrooms and Bioactive Plants in the Green Economy Value Chain Research Group, Institute of Biotechnology and Genetic Engineering, Chulalongkorn University, 254 Phayathai Road, Pathumwan, Bangkok, 10330, Thailand

* Corresponding author: sirasit.s@cmu.ac.th

Table S1. Response Surface Methodology (RSM) experimental design and corresponding experimental responses.

Run	Anaerobic effluent (%)	Molasses concentration (g/L)	Biomass (g/L)		Lipid yield (mg/L)		PUFA (%)	
			Predicted value	Experimental value	Predicted value	Experimental value	Predicted value	Experimental value
1	6.5	15	1.51	1.02±0.00	43.43	27.00±7.07	9.19	9.19±0.08
2	12.5	25.95	1.63	1.59±0.65	99.66	110.00±19.8	10.35	9.90±1.09
3	12.5	20	2.87	2.30±0.00	66.21	62.00±11.31	11.20	12.29±0.06
4	12.5	20	2.87	1.80±0.01	66.21	59.00±7.07	11.20	10.41±0.04
5	18.5	25	1.63	1.86±0.01	125.90	122.00±42.43	10.16	10.23±0.38
6	12.5	20	2.87	2.72±0.02	66.21	51.00±1.41	11.20	10.77±0.01
7	5.36	20	2.19	2.30±0.01	36.12	57.00±21.21	11.83	11.45±0.11
8	12.5	20	2.87	3.03±0.03	66.21	73.00±4.24	11.20	11.14±0.05
9	12.5	14.05	2.98	3.85±0.00	59.58	78.00±2.83	9.30	9.66±0.00
10	12.5	20	2.87	3.21±0.00	66.21	62.00±14.14	11.20	10.65±0.01
11	6.5	25	2.27	2.59±0.01	57.63	46.00±16.97	12.14	12.61±0.08
12	19.64	20	3.67	3.94±0.01	94.12	102.00±8.49	11.93	12.22±0.50
13	18.5	15	4.65	4.06±0.00	72.71	64.00±0.00	11.35	10.94±0.03
Optimal conditions	18.5	18.18	4.03	4.09±0.07	81.43	87.00±6.05	11.88	9.30±0.16

Table S2. Comparison of key nutritional lipid indices of *Chlorella* sp. G049 with other nutritional lipid sources.

Sources	Nutritional indices						References
	PUFA/SFA	AI	TI	h/H	HPI	UI	
Opisthonema oglinum	1.47	0.6	0.2	0.87	NA	NA	Fernandes *et al.* [1]
Salmo trutta	NA	0.64-0.72	0.21-0.30	NA	NA	NA	Dal Bosco *et al.* [2]
Pig	0.46-0.48	0.27-0.31	NA	NA	NA	NA	Alvarenga *et al.* [3]
Shrimp *Penaeus notialis*	NA	0.71–0.82	0.21–0.30	NA	NA	NA	Akintola [4]
Milk of sheep	0.06-0.09	1.76-2.72	1.00-1.47	0.50-0.68	NA	NA	Sinanoglou *et al.* [5]
Cheese of Comisana ewe	0.086–0.173	1.61-3.61	NA	NA	NA	NA	Bonanno *et al.* [6]
Cattle (*Nellore cattle*)	0.11–0.20	NA	NA	1.56–2.08	NA	NA	Correa *et al.* [7]
Lamb	0.13-0.37	0.49-0.52	1.1-1.15	NA	NA	NA	Majdoub-Mathlouthi *et al.* [8]
Sunflower oil	4.75-4.94	0.09-0.11	NA	NA	NA	NA	Filip *et al.* [9]
Lupinus albus	1.53-1.97	0.08-0.11	0.14-0.18	NA	NA	NA	Calabro *et al.* [10]
Dairy products	0.02-0.175	1.42-5.13	0.39-5.04	0.32-1.29	0.16-0.68	86-120	Chen and Liu [11]
Brown seaweed	0.20-1.38	0.53-1.99	0.46-1.60	1.26-1.90	NA	62.27-202.83	Chen *et al.* [12]
Red seaweed	0.14-2.12	0.38-2.87	0.52-5.75	2.09-4.22	NA	50.63-257.07	Paiva *et al.* [13]
Green seaweed	0.23-0.88	0.86-1.61	1.28-2.90	NA	NA	70.87-141.87	Kumar *et al.* [14]
Rhopalodia sp.	0.56	0.94	1.11	0.82	1.06	102.72	Pekkoh *et al.* [15]
Anomoeoneis sp.	0.37	0.68	1.15	0.57	1.46	104.01	Pekkoh *et al.* [15]
Algae blend macroalgae species	0.89-0.96	0.61-0.65	0.31-0.36	1.78-1.87	NA	NA	Mota *et al.* [16]
Commercial plant protein-based	1.01	0.58	0.30	1.94	NA	NA	Mota *et al.* [16]
Spirulina sp.	0.81	0.89	0.28	1.0	86.7	NA	Senila *et al.* [17]
Spirulina sp.	1.01	0.7	1.6	0.6	NA	NA	Conde *et al.* [18]
Spirulina sp.	0.278	1.128	2.261	0.442	0.886	59.737	Maneechote *et al.* [19]

Chlorella sp.	0.82	3.02	1.24	0.94	1.57	92.98	Sriket *et al*. [20]
Chlorella sp.	1.14	0.46	0.22	2.3	46.4	NA	Senila *et al*. [17]
Chlorella sp. G049	0.14±0.01	2.62±0.22	3.52±0.27	0.41±0.02	0.38±0.03	43.58±1.94	This study

Noted: NA is not available.

Table S3. Comparison of fatty acid profiles of lipids from *Chlorella* sp. G049 cultivated under optimal heterotrophic and various other conditions.

Conditions	Compositions (%)					References
	C16-C18	SFAs	UFAs	MUFAs	PUFAs	
Chlorella vulgaris cultivated under heterotrophic conditions	99.1	34.9	65.1	21.2	43.9	Couto *et al.* [21]
Chlorella sorokiniana in untreated dairy wastewater	63.75-73.83	38.84-44.88	22-33	1.3-1.8	21.8-32.36	Hamidian and Zamani [22]
C. vulgaris in Guillard's F2 medium under photoautotrophic mode	82.2	24.9	74.9	10.3	64.6	Conde *et al.* [18]
Tetraselmis chui in Guillard's F2 medium under photoautotrophic mode	94.3	33.6	66.3	14.5	51.8	Conde *et al*. [18]
C. sorokiniana with crude glycerol under heterotrophic mode	80-90	20-38	59-87	39-59	20-28	Mpesios *et al.* [23]
Scenedesmus sp. and *Limnothrix* sp. in domestic wastewater under mixotrophic mode	67.39-94.9	15.1-47.5	52.32-84.9	36.3-65.8	16.02-19.1	Devi *et al*. [24]
Chlorella pyrenoidosa using plant hormones in municipal wastewater	92.1	40.8	59.1	22.8	36.3	Wang *et al*. [25]
Graesiella emersonii GEGS21 under heterotrophic mode	90.51	36.66	63.35	37.05	26.3	Ki et al. [26]
C. sorokiniana in 39% poultry waste and 57% molasses under heterotrophic mode	90.48	39.93	59.28	23.3	35.98	Jareonsin *et al*. [27]
C. vulgaris culture in poultry slaughterhouse wastewater pH9-11 under photoautotrophic	69.90-86.09	42.99-68.19	27.90-60.90	7.95-37.05	19.95-23.85	Katırcıoğlu Sınmaz *et al.* [28]
Chlorella sp. G049 cultivated under heterotrophic with anaerobic wastewater and molasses	82.49±0.66	64.36±1.77	35.64±1.76	26.33±1.60	9.30±0.16	This study

Noted: SFAs refer to saturated fatty acids, UFAs to unsaturated fatty acids, MUFAs to monounsaturated fatty acids, and PUFAs to polyunsaturated fatty acids.

Table S4. Comparison of biodiesel fuel properties from *Chlorella* sp. G049 and various other algal strains.

Parameters	Algal stains								Biodiesel standards		
	Chlorella sorokiniana	*Chlorella vulgaris* MBFJNU-1	*Graesiella emersonii* GEGS21	*C. sorokiniana*	*C. sorokiniana* EAKI	*Chlorella pyrenoidosa*	*Scenedesmus* sp. DDVG I	*Chlorella* sp. G049	EN 14214	ASTM D6750	TH 2020
SV	202.71-203.71	NA	205.151	195.7-208.2	NA	NA	188.1-191.7	211.64±0.35	NA	<202	NA
IV	87.10-91.10	NA	88.398	80.1-96.0	55.07-72.80	67.7-80.12	67.96-71.47	40.64±1.77	≤120	NA	≤120
CN	52.73-53.50	56.34-56.41	53.015	51.9-54.5	57.48-59.07	48.2-48.7	58.6-60.02	62.95±0.36	≥51	≥47	≥51
DU	93.83-97.87	97-98	89.65	NA	NA	NA	66.74-68.51	44.94±1.93	NA	NA	NA
LCSF	4.65-5.55	NA	4.962	NA	NA	8.42-12.9	24.06-26.7	13.00±0.34	NA	NA	NA
CFPP	-1.87-0.95	NA	0.888	NA	NA	5.2-22	59.1-67.45	24.37±1.06	-20-5	NA	NA
HHV	39.44-39.48	NA	39.417	39.7-40.0	39.53-39.95	32.5-38.2	39.87-39.9	40.14±0.01	NA	NA	NA
OS	7.80-8.42	NA	7.075	NA	6.2-7.8	NA	6.31-6.6	15.27 ±0.23	≥6	>3	≥6
CP	2.68-3.72	6.91-7.04	12.587	NA	9.20-12.38	NA	-4.0	13.59 ±0.47	NA	NA	NA
ν	3.84-3.86	4.59	3.77	NA	4.69-4.85	2.602-3.14	4.6-4.8	6.72±0.00	3.5-5.0	1.9-6.0	<8
ρ	0.88	0.88	6.843	NA	0.876-0.877	NA	0.87	0.98±0.00	0.86-0.90	0.85-0.90	NA
APE	92.83-97.87	NA	NA	NA	NA	NA	NA	59.74±2.98	NA	NA	NA
BAPE	24.27-29.33	NA	NA	NA	NA	NA	NA	9.30±0.17	NA	NA	NA
References	Vyas *et al.* [29]	Xie *et al.* [30]	Ki *et al.* [26]	Mpesios et al. [23]	Hamidian and Zamani [22]	Jacob *et al.* [31]	Devi *et al.* [24]	This study	Pekkoh *et al.* [32]	Pekkoh *et al.* [32]	Pekkoh *et al.* [32]

Noted: NA indicates data not available.

Table S5. Comparison of pollutant removal efficiency with previous studies.

Microalgal strains	Effluents	Operating conditions	Pollutant removal (%)	References
Chlorella PY-ZU1	Anaerobic digestion effluent of food waste collected from household kitchen (COD 3108 mg/L, TN 2200 mg/L, TP 44 mg/L)	The effluent culture medium with an inoculum density of 0.1 g/L in continuous illumination of 6000 lux at 27°C under autotrophic condition for 10 days.	COD 68%<br>NH_3 99%<br>TP 99%	Cheng *et al.* [33]
Chlorella pyranoidosa FACHB-10	Furfural wastewater (COD 7692 mg/L, TP 2.87 mg/L)	FWW was diluted with distilled water (10-fold) with microalgal seeds 0.1 g/L and $NaNO_3$ 0.25 g/L under heterotrophic condition.	COD 81.17%<br>TN 75.44%<br>TP 96.68 %	Cheng *et al.* [34]
Chlorella sp.	Hydroponic effluent (COD 14490.70 mg/L, N-NO_3 145 mg/L, N-NH_4 120 mg/L, P-PO_4 256 mg/L)	Heterotrophic cultivation was conducted using 100% effluent supplemented with 12.2 g/L of glucose and 15 mg/L of IAA for 6 days.	COD 88.74%<br>NO_3 93.79%<br>NH_4 81.21%<br>PO_4 89.94%	Sriket *et al.* [20]
Chlorella sorokiniana	The simulated wastewater containing volatile fatty acids (VFAs) at 6 g/L from activated sludge	The initial density of 0.15 g/L. The total volume was 800 mL and placed in the dark for 6 days under heterotrophic condition.	TN 97.44%<br>TP 91.02%	Lu *et al.* [35]
Chlorella vulgaris	The centrate from dewatering of anaerobically digested sludge (COD 601.0 mg/L, N-NO_3 6.5 mg/L, N-NH_4 130 mg/L, TP 200.0 mg/L)	The volume of 350 mL<br>of wastewater was mixed with algal culture and incubated at 20 °C with a light intensity of 0.98 μW/cm^2 for 28 days under autotrophic condition.	COD 61.1%<br>N-NO_3 23.1%<br>NH_4 64.1%<br>TP 25.8%	AlMomani and Örmeci [36]
Chlorella sorokiniana SU-1	Dairy wastewater from the dairy farm of Tunghai University, Taichung, Taiwan. (COD: 4000 mg/L,	Dairy wastewater 50% diluted with BG-11 at 2 g/L inoculum size at continuously light intensity of 140 μmol m^{-2} s^{-1} under	COD 89.1%<br>TN 89.2%<br>TP 93.7%	Kusmayadi *et al.* [37]

	TN: 22 mg/L, TP 11.6 mg/L)	autotrophic condition for 12 days.		
Chlorella sp. G049	Anaerobic effluent 18.5% derived from food waste with molasses 18.18 g/L (COD: 3432 mg/L, N-NO_3: 7.5 mg/L, N-NH_4: 52.30 mg/L, P-PO_4: 12.5 mg/L, Total sugar: 2,150 mg/L<br>Na: 116.17 mg/L<br>Ca: 61.29 mg/L<br>Mg: 76.41 mg/L<br>S: 0.92 mg/L<br>Fe: 2.23 mg/L<br>Mn: 0.86 mg/L<br>Cu 0.04 mg/L<br>B: 0.42 mg/L	Heterotrophic cultivation at 150 rpm for 6 days.	COD 91.79%<br>N-NO_3 73.21%<br>N-NH_4 63.19%<br>P-PO_4 96.76%<br>Sugar consumption 57.07%	This study
